# miR-122 promotes hepatic lipogenesis via inhibiting the LKB1/AMPK pathway by targeting Sirt1 in non-alcoholic fatty liver disease

**DOI:** 10.1186/s10020-019-0085-2

**Published:** 2019-06-13

**Authors:** Jun-Ke Long, Wen Dai, Ya-Wen Zheng, Shui-Ping Zhao

**Affiliations:** 1 Department of Cardiovascular Medicine, The Second Xiangya Hospital of Central South University, No.139, Middle Renmin Road, Changsha, 410011 Hunan Province People’s Republic of China; 2 Department of Urological Organ Transplantation, The Second Xiangya Hospital of Central South University, Changsha, 410011 People’s Republic of China

**Keywords:** miR-122, Non-alcoholic fatty liver disease, Lipogenesis, Sirt1, AMPK pathway

## Abstract

**Background:**

Non-alcoholic fatty liver disease (NAFLD) is a common hepatic disease with an increasing prevalence but an unclear aetiology. This study aimed to investigate the functional implications of microRNA-122 (miR-122) in the pathogenesis of NAFLD and the possible molecular mechanisms.

**Methods:**

Both in vitro and in vivo models of NAFLD were generated by treating HepG2 and Huh-7 cells with free fatty acids (FFA) and by feeding mice a high-fat diet (HFD), respectively. HE and Oil Red O staining were used to examine liver tissue morphology and lipid deposition, respectively. Immunohistochemical (IHC) staining was used to examine Sirt1 expression in liver tissues. qRT-PCR and Western blotting were employed to measure the expression of miR-122, Sirt1, and proteins involved in lipogenesis and the AMPK pathway. Enzyme-linked immunosorbent assay (ELISA) was used to quantify triglyceride (TG) levels in HepG2 and Huh-7 cells and in liver tissues. The interaction between miR-122 and the Sirt1 gene was further examined by a dual luciferase reporter assay and RNA-immunoprecipitation (RIP).

**Results:**

NAFLD hepatic tissues and FFA-treated HepG2 and Huh-7 cells presented excess lipid production and TG secretion, accompanied by miR-122 upregulation, Sirt1 downregulation, and potentiated lipogenesis-related genes. miR-122 suppressed Sirt1 expression via binding to its 3′-untranslated region (UTR). Knockdown of miR-122 effectively mitigated excessive lipid production and suppressed the expression of lipogenic genes in FFA-treated HepG2 and Huh-7 cells via upregulating Sirt1. Furthermore, miR-122 knockdown activated the LKB1/AMPK signalling pathway.

**Conclusion:**

The inhibition of miR-122 protects hepatocytes from lipid metabolic disorders such as NAFLD and suppresses lipogenesis via elevating Sirt1 and activating the AMPK pathway. These data support miR-122 as a promising biomarker and drug target for NAFLD.

## Background

Nonalcoholic fatty liver disease (NAFLD) is a prevalent chronic disease worldwide and has become an emerging health threat in recent years (Spengler & Loomba, 2015). Epidemiological surveys have shown that the incidence of NAFLD is sharply increasing, mainly due to high-fructose diet ingestion and higher rates of obesity and diabetes (Dai et al., 2017a). The timely intervention of NAFLD is thus of critical importance for reducing public health burdens. Currently, major therapeutic strategies for NAFLD consist of lifestyle adjustment and medication that improve lipid homeostasis. However, the associated side effects of existing drugs, such as weight gain or higher long-term mortality, still restrain the medical treatment of NAFLD (Takahashi et al., 2015). Therefore, the identification of novel biomarkers is critical for both early diagnosis and the precise intervention of NAFLD.

microRNAs (miRNAs), which are small non-coding RNA molecules, can mediate metabolic homeostasis via unique post-transcriptional regulatory mechanisms (Rottiers & Naar, 2012). A role of miRNA in NAFLD has been suggested recently. Accumulating evidence has revealed the unique miRNA expression profiles that are tightly correlated with NAFLD conditions (Panera et al., 2014). For example, a recent study involving 47 NAFLD patients found that circulating levels of miR-122, miR-192 and miR-375 were upregulated and were positively correlated with disease severity (Pirola et al., 2015). In a rat NAFLD model, miR-122 has also been shown to be affected (Yamada et al., 2015). As one of the most abundant miRNAs in the liver,miR-122 was significantly upregulated in serum and was suggested as a potential biomarker for NAFLD in patients (Jampoka et al., 2018; Ye et al., 2018; Akuta et al., 2016). In particular, miR-122 upregulation was found to be directly associated with the severity of liver fibrosis in NAFLD patients (Miyaaki et al., 2014). It was thus speculated that miR-122 might function as a predictive factor for disease progression for NAFLD (Ceccarelli et al., 2013). However, the detailed molecular mechanism of miR-122 in NAFLD is still unclear.

Sirtuin 1 (Sirt1) is a nicotinamide adenine dinucleotide (NAD)-dependent deacetylase that has been implicated in NAFLD progression (Nassir & Ibdah, 2016). A previous study reported that Sirt1 expression was downregulated in liver tissues of a rat NAFLD model (Deng et al., 2007). Moreover, the knockout of the Sirt1 gene in hepatocytes suppressed fatty acid oxidation (Purushotham et al., 2009). D. Herranz et al. revealed that Sirt1 could protect liver tissues from ageing-related metabolic stress (Herranz et al., 2010). Mechanistically, Sirt1 regulates cellular metabolism via NAD-dependent histone deacetylation to affect transcriptional networks (Imai et al., 2000). Sirt1 also modulates the expression of various genes involved in glucose and lipid metabolism (Li, 2013). Upstream of Sirt1, its expression is regulated by various non-coding RNAs, such as miR-34a (Li et al., 2015). However, the relationship between Sirt1 and miR-122 in NAFLD remains elusive.

In this study, we performed both in vitro and in vivo experiments to examine the function of miR-122 on Sirt1-mediated lipid metabolism in NAFLD pathogenesis. We found that miR-122 was upregulated both in cell lines and in an animal model of NAFLD; by contrast, Sirt1 expression was downregulated. A mechanistic study revealed that miR-122 directly bound to the 3’-UTR of Sirt1 to suppress its expression. Knockdown of miR-122 decreased the expression of lipogenic genes but activated the AMPK signalling pathway, which further suppressed lipid production and triglyceride (TG) secretion in hepatocytes. Our results thus provide the novel insight into NAFLD pathogenesis and highlight the potency of miR-122 as both a biomarker and therapeutic target for NAFLD in the future.

## Methods

### Cell culture and treatment

The human hepatocyte cell lines HepG2 and Huh-7 were obtained from the American Type Culture Collection (ATCC, US) and cultured in a humidified incubator at 37 °C with 5% CO_2_ using Dulbecco’s Modified Eagle Medium (DMEM) (Gibco, US), which was supplemented with 1% penicillin and streptomycin (Invitrogen, US) and 10% foetal bovine serum (FBS) (Gibco, US). To establish the in vitro NAFLD cell model, HepG2 and Huh-7cells were cultured in the presence or absence of 1 mM free fatty acids (FFA, containing oleic acid and palmitic acid at a 2:1 volume ratio) for 24 h and then used for the indicated assays.

### NAFLD mouse model

The NAFLD mouse model was developed according to previously reported protocols (Soares e Silva et al., 2015). In brief, a total of 16 C57BL/6 mice (males, 8 weeks old) were purchased from SJA Laboratory Animal Corp (Changsha, China) and were randomly assigned to the high-fat diet (HFD) or the standard chow diet (SCD) group (*n* = 8 in each group). Mice were then fed the indicated food chow for 8 weeks to establish the NAFLD model. All animal experimental procedures followed the guidelines of the Ethical Committee of the Second Xiangya Hospital, Central South University.

### ELISA assay

The TG contents in HepG2 and Huh-7 cells or liver tissues isolated from the NAFLD mouse model were measured with an ELISA assay. Briefly, HepG2 and Huh-7 cells were pre-treated with FFA, and then the cell lysates and the supernatant were collected for TG quantification. For NAFLD mice, liver tissues were collected after sacrifice and then homogenized and lysed in 5% NP-40/ddH_2_O solution. After centrifugation, the supernatant was collected for subsequent TG quantification. The concentration of TG was measured using ELISA kits (Abcam, UK) according to the manufacturer’s instructions.

### Cell transfection

The sequences of the shRNA targeting Sirt1 (shSirt1) and the negative control shRNA (shNC) were 5′- CCGGGATGATCAAGAGGCAATTAATCTCGAGATTAATTGCCTCTTGATCATCTTTTTG-3′ and 5′-CCGGCCTAAGGTTAAGTCGCCCTCGCTCGAGCGAGGGCGACTTAACCTTAGGTTTTTG-3′, respectively. shSirt1 and shNC were subcloned into the pSicoR plasmid vector, and sequence-verified plasmids were then used for subsequent transfection. miR-122 mimics, inhibitors and negative control miRNAs were purchased from GenePharma (Shanghai, China). Cell transfection and cotransfection were conducted with Lipofectamine 2000 (Invitrogen, USA) according to the manufacturer’s instruction. In brief, 5 μL of Lipofectamine-2000 reagent was diluted into 0.25 mL of OPTI-MEM medium, while 5 μL of miR-122 mimics/inhibitor/negative control miRNA (100 pmol) or 4.0 μg of pSicoR-Sirt1/pSicoR plasmid was added to another 0.25 mL aliquot of OPTI-MEM medium. Then, they were incubated separately for 5 min at room temperature. After that, they were combined in one tube, gently inverted and incubated for 20 min. Subsequently, 0.5 mL of the mixture was added to the HepG2 and Huh-7 cells drop wise. The plate was then swirled several times to ensure that the mixture was distributed evenly. After culturing for 6 h, the medium was replaced with fresh DMEM medium containing 10% FBS. The cells were then cultured for another 48 h before the subsequent experiments.

### Dual luciferase reporter assay

A dual luciferase reporter assay was employed to examine the interaction between Sirt1 and miR-122 in HepG2 and Huh-7 cells according to a previously reported method (Hu et al., 2014). Specifically, wild-type and mutant (without miR-122 binding sites) three prime untranslated regions (3′-UTRs) from Sirt1 mRNAs were cloned and inserted downstream of the *luc2* firefly luciferase in the pmiRGLO vector (Promega, US), which was then co-transfected with miR-122 mimics into HepG2 and Huh-7 cells. The constitutively expressed *Renilla* luciferase in the pmiRGLO vector provided a normalization reference. After 48 h of transfection, the HepG2 and Huh-7 cells were lysed and subjected to luciferase activity measurement with the Dual Luciferase Assay Kit (Promega, US) according to the manufacturer’s instructions. The firefly and *Renilla* luciferase activities were determined by a plate reader (NEO, Bio-Tek, USA) and normalized to the *Renilla* luciferase data.

### RNA immunoprecipitation (RIP)

We used an RIP assay to directly confirm the interaction between miR-122 and Sirt1 according to a method in a previous report (Chen et al., 2018). Briefly, HepG2 and Huh-7 cells were lysed in 300 μL of lysis buffer supplemented with RNase inhibitor and complete protease inhibitor for 20 min on ice, and then the cell lysate was centrifuged for 10 min at 4 °C. The supernatant was collected and pre-cleared with protein A-Sepharose beads. Next, the supernatant was incubated with mouse anti-human Ago2 antibody (Cell Signaling Technology, USA) or negative control antibody (normal mouse IgG, Cell Signaling Technology, USA) for 4 h, followed by the addition of protein A/G sepharose beads and incubation for 2 h. The beads were then rinsed with NT2 buffer supplemented with RNase inhibitor and complete protease inhibitors, and bead-captured protein-RNA complexes were digested using DNase I and proteinase K and were then eluted by NT2 buffer. RNA was finally extracted by the phenol-chloroform method and quantified by qRT-PCR. The primers used for amplifying the Sirt1 were as follows: forward: 5′-TTTGTCAGAGTTGCCACCCA-3′; reverse: 5′-GCCGCCTACTAATCTGCTCC-3′.

### RNA extraction and quantitative real-time PCR (qRT-PCR)

qRT-PCR was used to quantify the relative expression levels of the miR-122, Sirt1, sterol regulatory element-binding protein 1(SREBP1), stearoyl-CoA desaturase 1(SCD1), acetyl-coA carboxylase (ACC1), fatty acid synthase (FASN) and apolipoprotein A5 (ApoA5) genes in both mouse liver tissues and cultured hepatocytes. In brief, total RNA including small RNA was extracted from liver tissues, HepG2 and Huh-7 cells using the miRNeasy Mini Kit (Qiagen, Germany) according to the manufacturer’s instructions. The first-strand cDNA was synthesized via in vitro reverse transcription (RT) from total RNA by using the QuantiTect Reverse Transcription Kit (Qiagen, Germany) or from small RNA by using the miScript II RT Kit (Qiagen, Germany). qRT-PCR was then performed on an Applied Biosystems™ QuantStudio™ 6 Flex Real-Time PCR System (Thermo Fisher Scientific, Carlsbad, CA, USA) with the SYBR Green method. PCR was performed under the following conditions: 95 °C for 10 min, followed by 40 cycles each of 95 °C for 15 s, 60 °C for 20 s and 72 °C for 40 s. The Ct values were calculated, and the relative expression level was quantified by the 2^-△△Ct^ approach using β-actin and U6 as the normalization references for mRNA and miRNA, respectively. Each sample was tested in triplicate for statistical analysis. The primers used in qRT-PCR are listed in Table 1.

**Table 1:** Primers used for qRT-PCR analysis

Genes	Primer sequences (5′-3′)
hsa(mmu)-miR-122	F: 5′-AGCGTGGAGTGTGACAATGG-3’
	R: 5′-GTCGTATCCAGTGCAGGGTCCGAG GTATTCGCACTGGATACGACCAAACA-3’
hsa(mmu)-U6	F: 5′-CTCGCTTCGGCAGCACA-3’
	R: 5′-AACGCTTCACGAATTTGCGT-3’
hSirt1	F: 5′-TGCCGGAAACAATACCTCCA-3’
	R: 5′-AGACACCCCAGCTCCAGTTA-3’
hSREBP1	F: 5′-GGAGCCATGGATTGCACTTTCG-3’
	R: 5′-GCTCAGGAAGGCTTCAAGAGAG-3’
hFASN	F: 5′-TATGAAGCCATCGTGGACGG-3’
	R: 5′-GAAGAAGGAGAGCCGGTTGG-3’
hSCD1	F: 5′-CTTGCGATATGCTGTGGTGC-3’
	R: 5′-AAGTTGATGTGCCAGCGGTA-3’
hACC1	F: 5′-CAAGGTCAGCTGGTCCACATG-3’
	R: 5′-GTGGAATACCTTCTGCCCTAGC-3’
hApoA5	F: 5′- GACCAGGAGACTGAGGAGGT-3’
	R: 5′-TTGCTCAGAACCTTGCCACT-3’
hβ-actin	F: 5′- CCTCGCCTTTGCCGATCC-3’
	R: 5′- GGATCTTCATGAGGTAGTCAGTC − 3’
mSirt1	F: 5′-GACGCTGTGGCAGATTGTTA-3’
	R: 5′-GGAATCCCACAGGAGACAGA-3’
mSREBP1	F: 5′-CCACAATGCCATTGAGAAGCG-3’
	R: 5′-CTGACACCAGGTCCTTCAGTG-3’
mFASN	F: 5′-TTGCTGGCACTACAGAATGC-3’
	R: 5′-AACAGCCTCAGAGCGACAAT-3’
mSCD1	F: 5′-CACACCTTCCCCTTCGACTA-3’
	R: 5′-TGACTCCCGTCTCCAGTTCT-3’
mACC1	F: 5′-CTTGGAGCAGAGAACCTTCG-3’
	R: 5′-ACTTCCCGACCAAGGACTTT-3’
mApoA5	F: 5′-AGAAGCTGGCACAGGAGAAC-3’
	R: 5′-AGCTGAGCCTTGGTGTCTTC-3’
mβ-actin	F: 5′-GTCGTACCACAGGCATTGTGATGG-3’
	R: 5′-GCAATGCCTGGGTACATGGTGG-3’

*Note:* The primers were manufactured by Nanjing Genescript Co. Ltd. (Nanjing, P.R China)

### Western blotting

Western blotting was used to measure the expression levels of Sirt1, liver kinase B1 (LKB1), AMP-activated protein kinase (AMPK) and p-AMPK proteins both in cell lines and in mouse liver tissues. In brief, cells or tissues were lysed and homogenized in RIPA buffer (50 mM Tris-HCl, 150 mM NaCl, 1% NP-40, 0.5% sodium deoxycholate, and 0.1% SDS, pH 7.4) supplemented with 1 × protease inhibitor cocktail, and the protein contents in the cell lysates were quantified by a BCA kit (Beyotime, China). Approximately 30 μg of protein sample was subjected to SDS-PAGE separation and was then transferred to a PVDF membrane (Millipore Corp, Bedford, MA). After blocking with 5% BSA in Tris-buffered saline with 0.1% Tween 20 (TBST), the PVDF membrane was rinsed and incubated with primary antibody (rabbit anti-Sirt1 antibody at 1:1000, rabbit anti-LKB1 antibody at 1:2000, rabbit anti-AMPK antibody at 1:1500, and rabbit anti-p-AMPK antibody and rabbit anti-GAPDH antibody at 1:1000, Cell Signaling Technology, USA) at 4 °C overnight. After vigorous washing with TBST 3 times, the PVDF membrane was then incubated with goat anti-rabbit IgG-HRP antibody (1:5000, Cell Signaling Technology, USA) for 1 h at room temperature. After washing with TBST, the membrane was then incubated with ECL substrate, and images were captured by a ChemiDoc MP imaging system (Bio-Rad, USA).

### Oil red O staining

Oil Red O staining was applied to assess lipid droplet formation in HepG2 and Huh-7 cells and liver tissues according to a previously described method (Chu et al., 2014). First, HepG2 and Huh-7 cells were pre-cultured on coverslips and were then washed with PBS and fixed in 10% formalin for 5 min. After fixation, the cells were briefly rinsed in isopropanol and incubated with Oil Red O reagent for 30 min. The stained cells were then washed in distilled water and counter-stained with haematoxylin for 1 min. The mouse liver tissues were first fixed in 4% paraformaldehyde and then embedded in paraffin and sectioned into slices with 6-μm thickness. These sections were then de-waxed in xylene and rehydrated. After rinsing with PBS, the tissue sections were incubated in Oil Red O reagent for 30 min, followed by haematoxylin counter-staining for 1 min. After washing and dehydration, the Oil Red O and haematoxylin-stained sections were mounted for fluorescence microscope (IX-51, Olympus) for imaging. Five randomly selected images were taken for each sample. Data were collected from at least three independent experiments.

### HE staining of liver tissues

Haematoxylin-eosin (HE) staining was performed to examine liver tissue morphology according to standard protocols. In brief, liver tissues were fixed with 4% paraformaldehyde (PFA) overnight. The fixed tissue samples were then embedded in paraffin and sectioned into slices with 6 μm thickness, which were then dehydrated with different concentrations of ethanol and xylol followed by brief washing and staining of cell nuclei with 5% haematoxylin solution for 10 min. After rinsing in distilled water for 5 min, the stained samples were incubated in 0.1% HCl-ethanol for 30 s. The samples were then counterstained with eosin solution for 2 min. After washing and dehydration, the HE-stained sections were mounted for fluorescence microscope (IX-51, Olympus) for imaging.

### Immunohistochemistry (IHC)

IHC was used to measure the expression of Sirt1 protein in mouse liver tissues. In brief, liver tissues were fixed with 4% PFA and embedded in paraffin. The samples were then sectioned into slices with a thickness of 6 μm, which were then attached to slides, washed and incubated with 3% H_2_O_2_ in methanol for 10 min to block endogenous peroxidase activity. Afterwards, these sections were blocked and then incubated with a primary antibody against Sirt1 (1:500, Abcam) at 4 °C overnight. After washing with PBS, the sections were incubated with secondary antibody for 1 h at room temperature. Finally, the sections were washed and incubated with DAB substrate for visualization. After washing and dehydration, the sections were sealed with coverslips and subjected to fluorescence microscopy (IX-51, Olympus) for imaging.

### Statistical analysis

All data are presented as the mean ± standard deviation (SD). Student’s *t*-test was used to compare differences between two groups, while one-way analysis of variance (ANOVA) was performed to compare differences among multiple groups, which was followed by Tukey’s post hoc comparison test. Statistical significance was indicated when *p* < 0.05. All statistical analyses were performed using SPSS18.0 software.

## Results

### Elevated miR-122 expression and decreased Sirt1 expression in the NAFLD mouse model

**Fig. 1:**
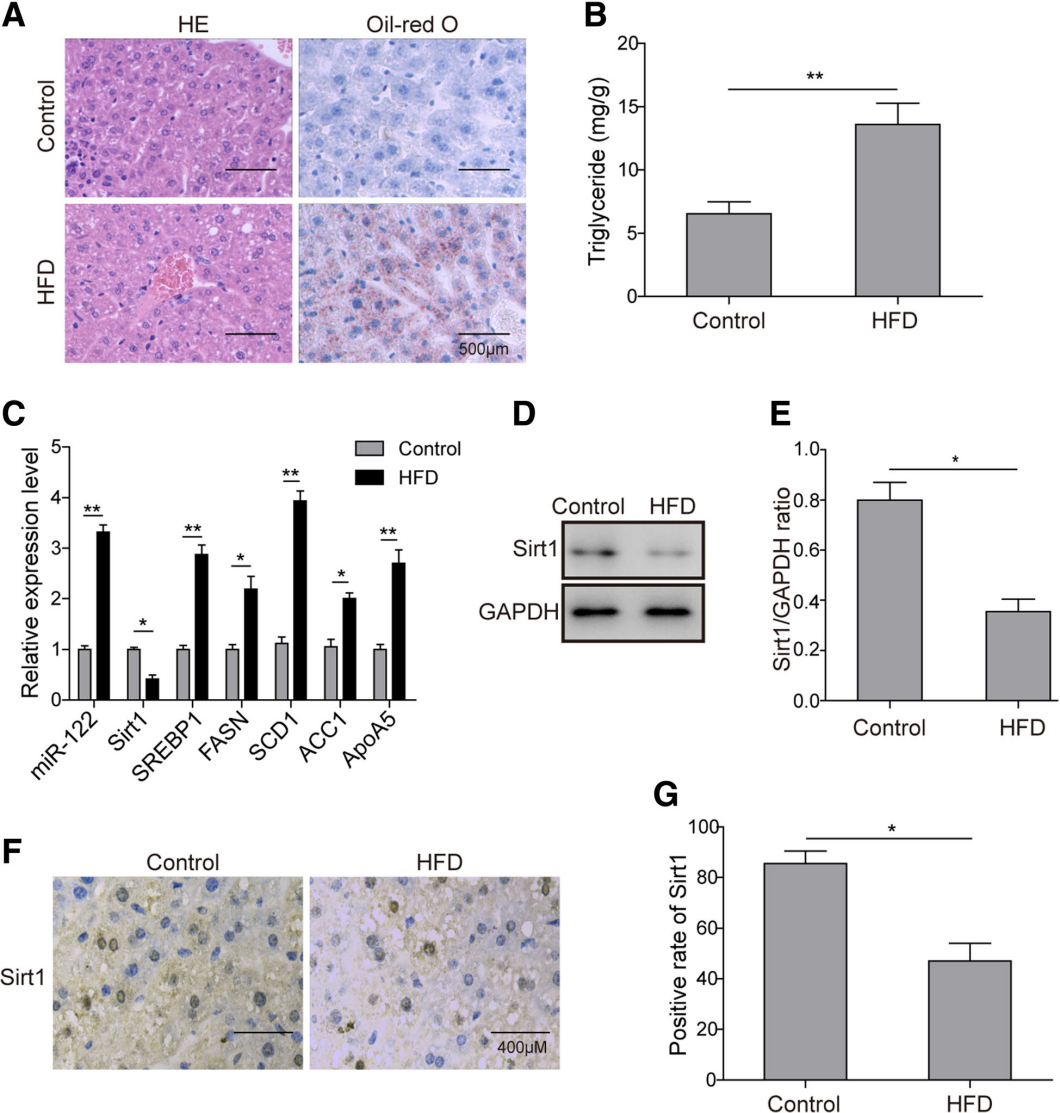
Lipid pathology and altered gene expression in NAFLD mice. **a** HE staining (left) and Oil Red O staining (right) showing lipid accumulation in liver tissues. **b** Hepatic TG levels were assessed by ELISA. **c** The relative expression levels of miR-122, Sirt1 and lipogenesis-related genes in control diet- and high-fat diet (HFD)-fed mice were detected by qRT-PCR. **d** The expression level of Sirt1 was measured by Western blotting. **e** Quantification of the Western blotting results in d. **f**, **g** Representative images (**f**) and thepositive rate (**g**) of Sirt1 expression in the liver tissues of NAFLD mice as revealed by IHC staining. All the results are shown as the mean ± SD (*n* = 3), which are representative of three independent experiments performed in triplicate. *p < 0.05 and **p < 0.01

To explore the relationship between Sirt1 and miR-122 in NAFLD, we first generated a mouse model of NAFLD using HFD feeding for 8 weeks. HE staining of liver tissues showed that there were increased hepatocyte volumes and more dispersed lipid vacuoles and compressed liver sinusoids in NAFLD mice compared to those in control mice (Fig. 1a). Consistently, Oil Red O staining results showed remarkable deposition of lipid droplets within the liver in NAFLD mice (Fig. 1a). Furthermore, an elevated TG secretion level was also identified in NAFLD mice (Fig. 1b). In addition, we further investigated the expression of miR-122 in mouse liver tissues. To our surprise, we found that it was upregulated in the liver of NAFLD mice (Fig. 1c). In addition, we examined multiple genes involved in hepatic lipogenesis, such as Sirt1, SREBP1, FASN, SCD1, ACC1 and ApoA5, by qRT-PCR. The data showed that only Sirt1 expression was reduced, while the expression of the other genes was elevated in NAFLD mice (Fig. 1c). Moreover, both Western blotting (Fig. 1d and e) and IHC staining (Fig. 1f and g) confirmed the decreased Sirt1 protein expression in liver tissues of NAFLD mice. In summary, these experiments revealed that Sirt1 was downregulated, while miR-122 and other lipogenic genes were all upregulated in the livers of NAFLD mice.

### FFA induces miR-122 upregulation and lipogenesis in hepatocytes

**Fig. 2:**
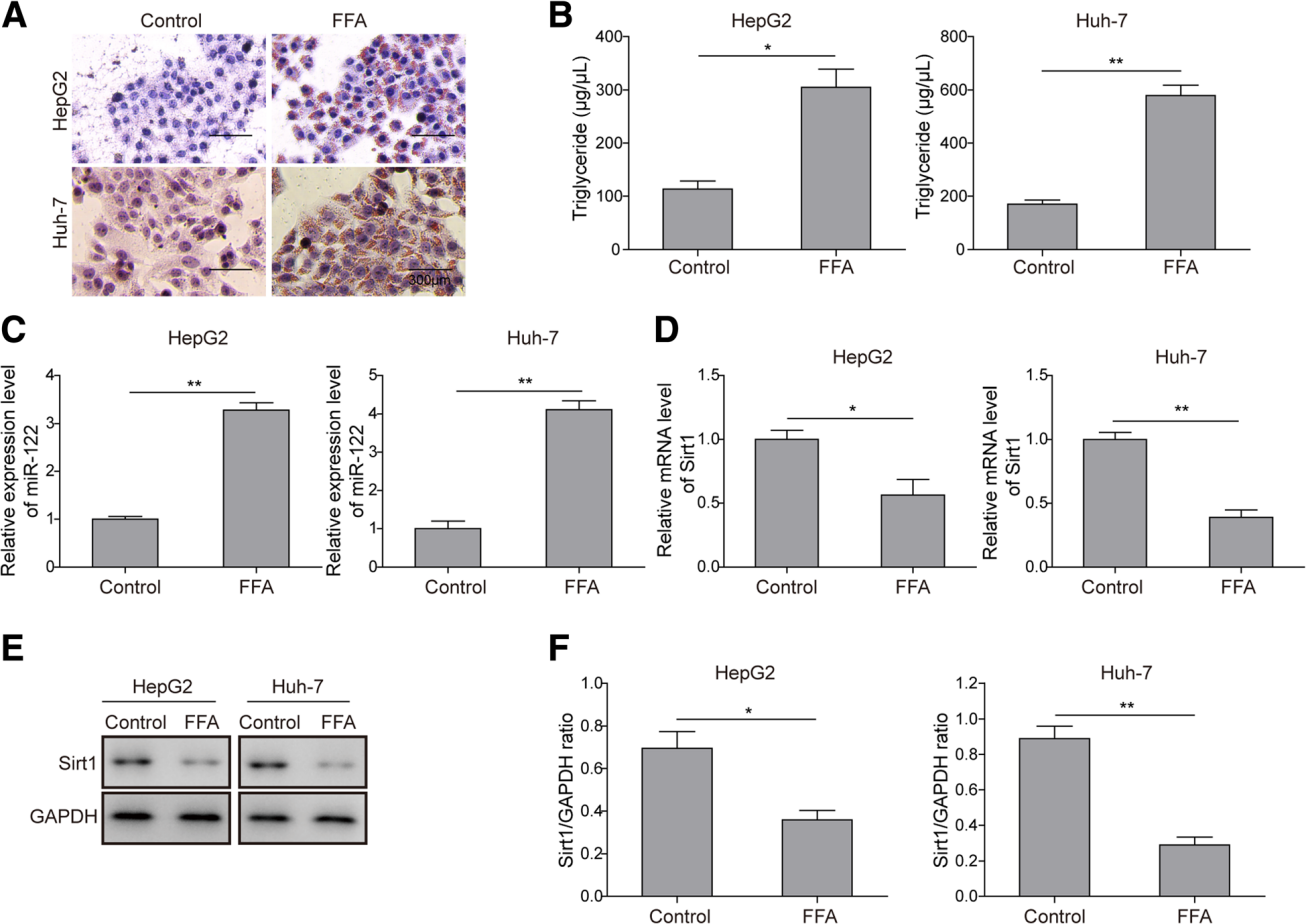
Effects of FFA on lipogenesis and miR-122 expression in hepatocytes. HepG2 or Huh-7 cells were pre-treated with 1 mM FFA for 24 h. **a** Oil Red O staining for lipid droplets in HepG2 or Huh-7 cells. **b** The secretion of TG in HepG2 or Huh-7 cells was measured by ELISA. **c**, **d** The relative expression of miR-122 (**c**) and Sirt1 (**d**) in HepG2 or Huh-7 cells was determined by qRT-PCR. **e** The expression level of Sirt1 in HepG2 or Huh-7 cells was measured by Western blotting. **f** Quantification of the corresponding Western blotting results in e. All the results are shown as the mean ± SD (*n* = 3), which are representative of three independent experiments performed in triplicate. *p < 0.05 and **p < 0.01

To examine the molecular pathway downstream of miR-122, we used two commonly used hepatocyte cell lines, HepG2 and Huh-7, for the in vitro cellular experiments. To mimic the NAFLD phenotype, HepG2 and Huh-7 cells were treated with FFA for 24 h. As expected, Oil Red O staining showed that FFA treatment remarkably induced lipid deposition inside cells (Fig. 2a). Moreover, FFA challenge robustly elevated TG secretion from HepG2 and Huh-7 cells (Fig. 2b). Consistent with NAFLD mice, both cell lines displayed elevated miR-122 expression but decreased Sirt1 expression as evidenced by qRT-PCR (Fig. 2c and d). In addition, Western blotting results confirmed that the protein level of Sirt1 was attenuated in FFA-treated HepG2 and Huh-7 cells (Fig. 2e and f). Overall, these data indicated that miR-122 might be involved in regulating FFA-induced lipogenesis and was negatively correlated with Sirt1 expression.

### miR-122 suppressesSirt1 expression via binding to the 3′-UTR of Sirt1

**Fig. 3:**
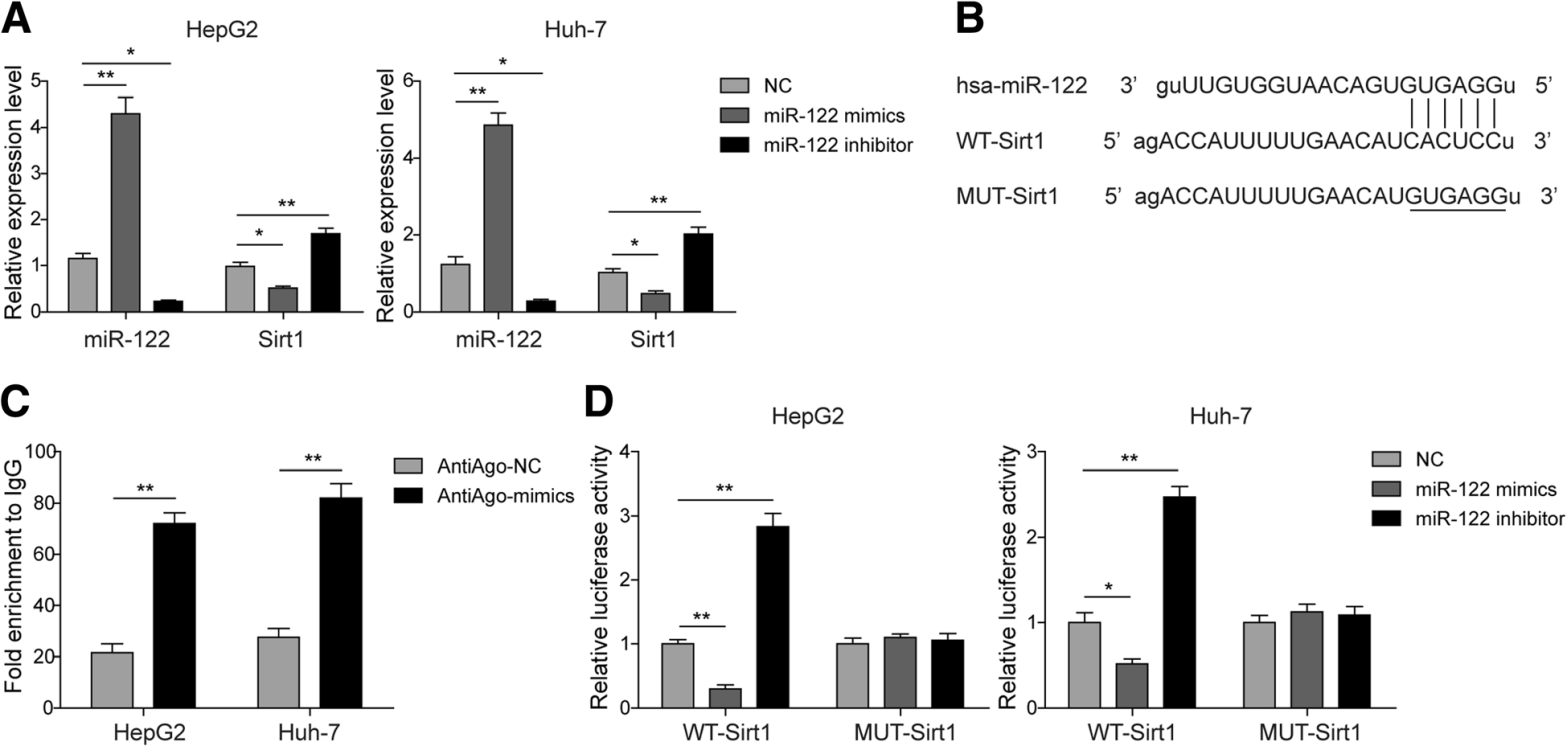
Direct interaction between miR-122 and Sirt1. **a** The relative expression levels of miR-122 and Sirt1 in HepG2 or Huh-7 cells transfected with miR-122 mimics or inhibitor. **b** Sequences of the putative binding sites of miR-122 in the 3′-UTR of Sirt1. **c** An RIP assay was performed to verify the interaction between miR-122 and Sirt1, and the Sirt1 mRNA level was measured by qRT-PCR. **d** A dual luciferase activity assay was implemented to confirm the direct association between miR-122 and the 3′-UTR of Sirt1. All the results are shown as the mean ± SD (*n* = 3), which are representative of three independent experiments performed in triplicate. *p < 0.05 and **p < 0.01

To further study the relationship between Sirt1 and miR-122, we next transfected HepG2 and Huh-7 cells with the miR-122 mimics, the miR-122 inhibitor or the negative control (NC). qRT-PCR results showed that the miR-122 mimics remarkably decreased Sirt1 expression; however, the miR-122 inhibitor potently suppressed miR-122 expression but elevated Sirt1 expression in both HepG2 and Huh-7 cells (Fig. 3a). These data indicated that the Sirt1 gene was possibly negatively regulated by miR-122 in hepatocytes. To further confirm the association of these factors, we performed a bioinformatic prediction and found putative binding sites of miR-122 in the 3′-UTR of Sirt1 (Fig. 3b). To verify this prediction, we then executed RIP assay, and the results strongly supported the direct interaction between miR-122 and Sirt1, which was suggested by the significant enrichment of Sirt1 mRNA when miR-122 was overexpressed in HepG2 and Huh-7 cells (Fig. 3c). Furthermore, we applied a dual luciferase assay to measure the binding between the miR-122 mimics or inhibitors and the 3′-UTR of Sirt1. Consistently, the results showed that the miR-122 mimics, but not the NC, clearly repressed luciferase activity, and this suppression was completely abolished when the binding sites in the 3′-UTR of Sirt1 were mutated (Fig. 3d). Overall, these results indicated that miR-122 participated in the modulation of Sirt1 via binding to the 3′-UTR of Sirt1.

### Knockdown of miR-122 alleviates lipid deposition by directly upregulating Sirt1 in hepatocytes

**Fig. 4:**
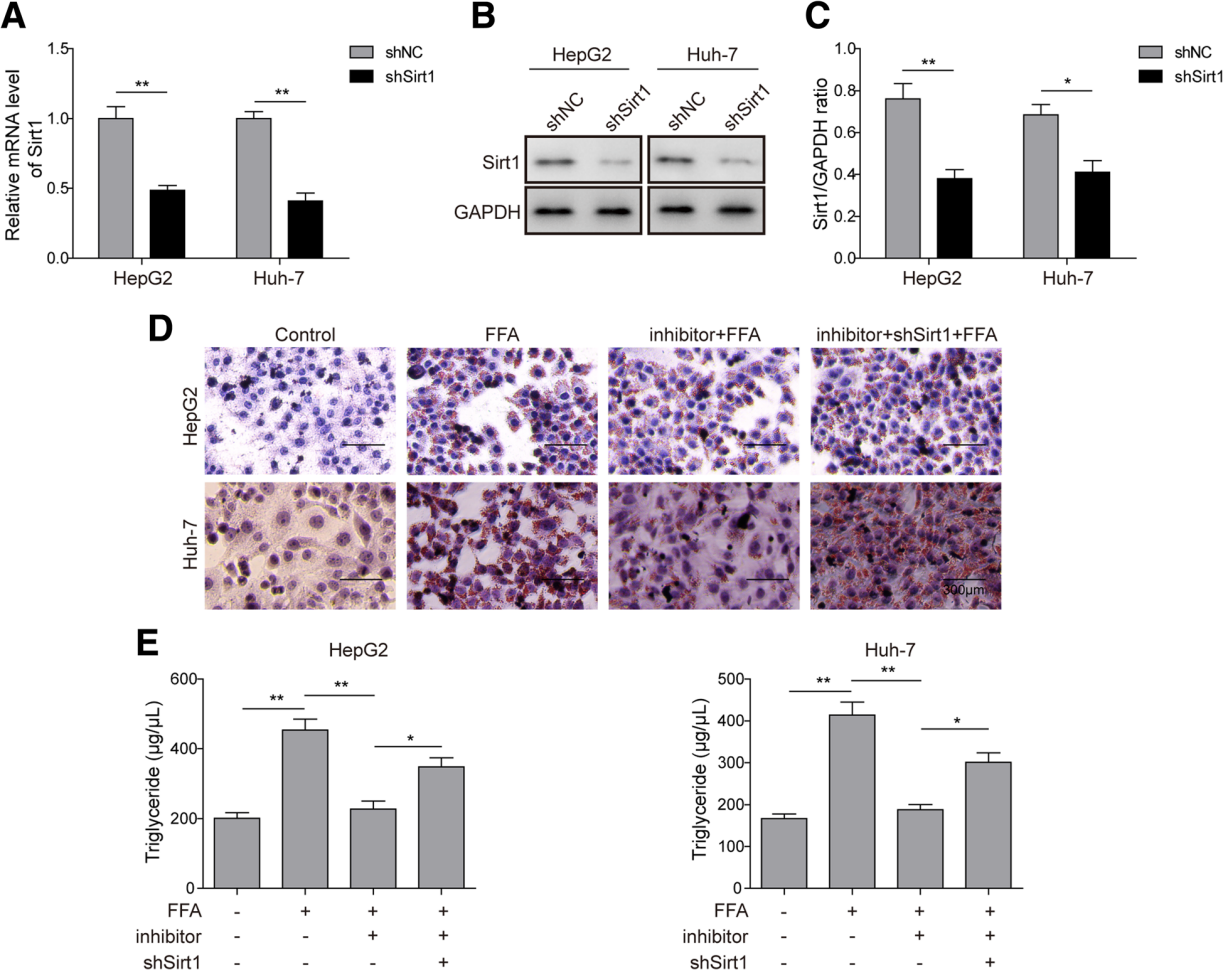
Suppression of lipid accumulation by the miR-122 inhibitor via the elevation of Sirt1 expression. **a** The relative expression level of Sirt1 was assessed by qRT-PCR in HepG2 or Huh-7 cells transfected with shSirt1. **b** The protein level of Sirt1 was assessed by Western blotting in HepG2 or Huh-7 cells transfected with shSirt1. **c** Quantification of the corresponding Western blotting results in b. **d** Oil Red O staining was performed to examine lipid accumulation in HepG2 or Huh-7 cells transfected with miR-122 inhibitor and/or shSirt1. **e** TG secretion levels in all groups of HepG2 or Huh-7 cells were measured by ELISA. All the results are shown as the mean ± SD (*n* = 3), which are representative of three independent experiments performed in triplicate. *p < 0.05 and **p < 0.01

After revealing the association between miR-122 and Sirt1, we next investigated the influence of this interaction on lipid metabolic disorder in hepatocytes by knocking down miR-122 or Sirt1. First, we transfected shSirt1 into HepG2 or Huh-7 cells, and subsequent qRT-PCR and Western blotting results showed that the expression level of Sirt1 was remarkably decreased (Fig. 4a-c), which indicated that the Sirt1 knockdown was successful. Afterward, we found that miR-122 knockdown by transfecting miR-122 inhibitor into HepG2 or Huh-7 cells could potently decrease lipid deposition induced by FFA treatment; nevertheless, this inhibitory effect was abrogated by the co-transfection of shSirt1 (Fig. 4d). Moreover, miR-122 knockdown also restrained FFA-induced TG secretion; however, additional Sirt1 silencing could reverse this phenotype (Fig. 4e). These results evidenced that miR-122 inhibition could protect hepatocytes from excessive lipid deposition via the elevation ofSirt1 expression.

### miR-122 modulates lipogenic gene expression via targeting ofSirt1

**Fig. 5:**
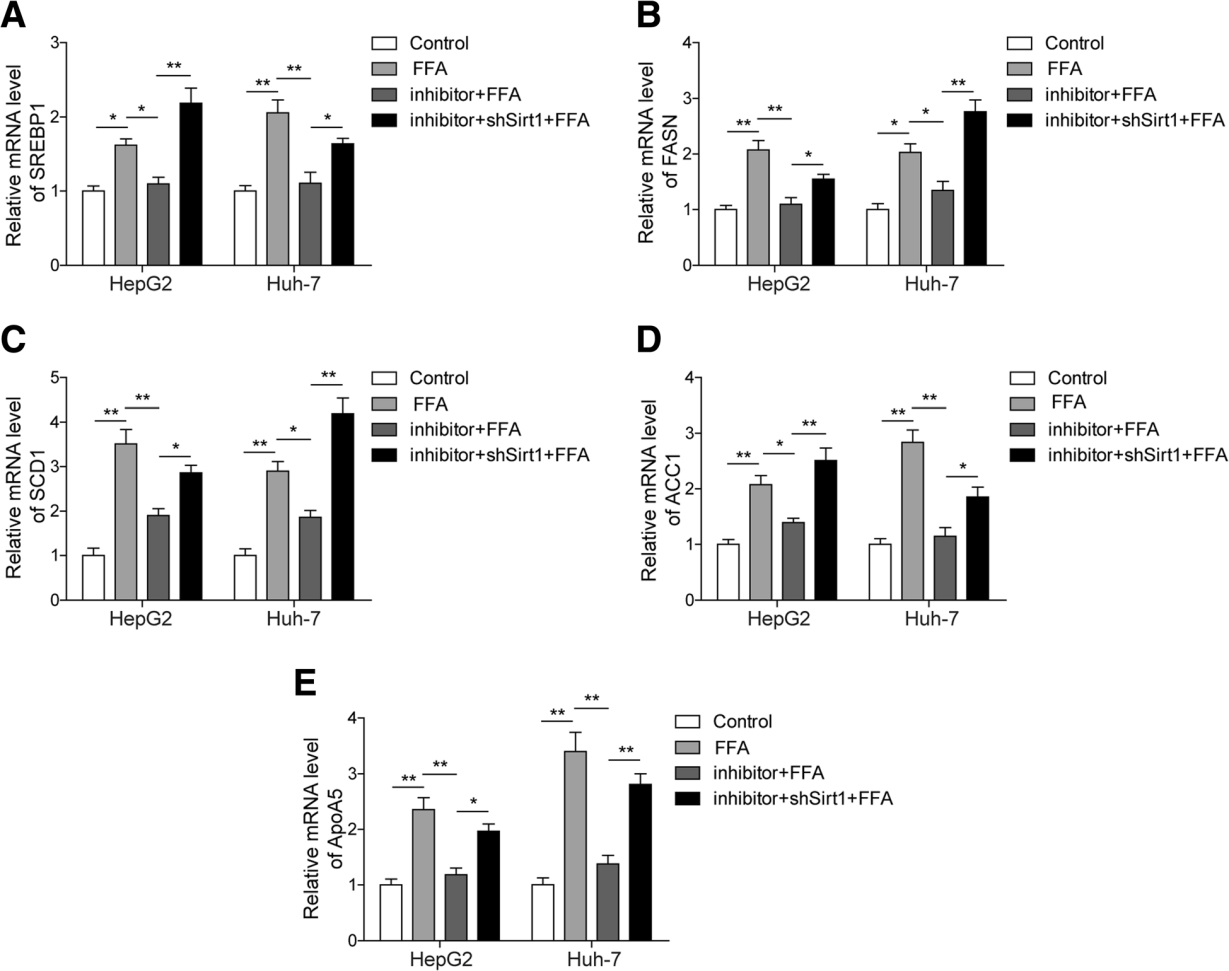
The expression of lipogenesis-associated genes was regulated by miR-122 and Sirt1. HepG2 and Huh-7 cells were pre-transfected with miR-122 inhibitor and/or shSirt1 and were then treated with 1 mM FFA for 24 h. **a**-**e** The relative expression levels of SREBP1 (**a**), FASN (**b**), SCD1 (**c**), ACC1 (**d**) and ApoA5 (**e**) in HepG2 and Huh-7 cells were detected by qRT-PCR. All the results are shown as the mean ± SD (*n* = 3), which are representative of three independent experiments performed in triplicate. *p < 0.05 and **p < 0.01

To ascertain the underlying molecular mechanism of the regulation of intracellular lipid metabolism by miR-122, we measured the expression levels of major lipogenic genes, such as SREBP1, FASN, SCD1, ACC1 and ApoA5, in FFA-treated cells transfected with miR-122 inhibitor and/or shSirt1. qRT-PCR results showed that FFA treatment elevated the expression of these genes; however, their expression levels were suppressed by miR-122 inhibitor (Fig. 5). In addition, the co-transfection of miR-122 inhibitor and shSirt1 reinstated the expression of lipogenic genes at levels similar to those in cells treated solely with FFA (Fig. 5). These results clearly illustrated that miR-122 repressed Sirt1 expression, which then led to the upregulation of lipogenic genes and the consequential overproduction oflipids or steatosis in hepatocytes. Therefore, knockdown of miR-122 could effectively downregulate lipogenic genes via elevating Sirt1 expression in hepatocytes.

### Knockdown of miR-122 activates the LKB1/AMPK signalling pathway via enhancing Sirt1 expression

**Fig. 6:**
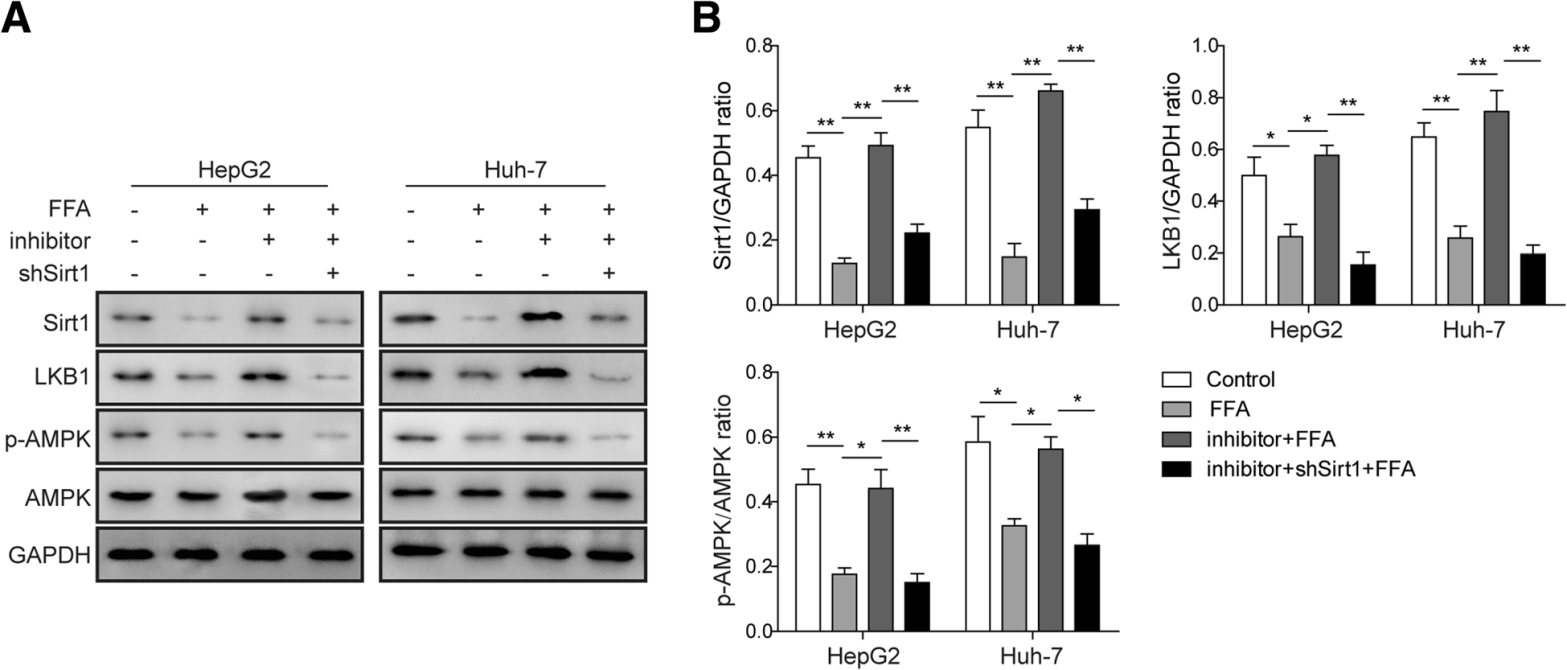
Downregulation of the LKB1/AMPK signalling pathway by the miR-122-Sirt1 axis. **a** The expression of Sirt1, LKB1, AMPK and the phosphorylation of AMPK (p-AMPK) were measured by Western blotting in HepG2 or Huh-7 cells that were transfected with miR-122 inhibitor and/or shSirt1. **b** Quantification of the corresponding Western blotting results in a. All the results are shown as the mean ± SD (*n* = 3), which are representative of three independent experiments performed in triplicate. *p < 0.05 and **p < 0.01

Previous studies have shown that the LKB1/AMPK pathway was implicated in the progression of NAFLD (Santamarina et al., 2015). Moreover, AMPK has been found to augment Sirt1 activity via elevating cellular NAD^+^ levels (Canto et al., 2009), while Sirt1 overexpression also led to the activation of LKB1 and AMPK (Lan et al., 2008). To investigate whether the LKB1/AMPK pathway is required for the regulation of lipogenesis by miR-122 through Sirt1,we performed Western blotting to assess the influence of FFA treatment and miR-122 knockdown on the activation of the LKB1/AMPK pathway. As expected, the result showed that FFA administration repressed the expression of Sirt1 and LKB1 as well as the phosphorylation of AMPK (p-AMPK) in HepG2 or Huh-7 cells. Nevertheless, the knockdown of miR-122 robustly upregulated LKB1 expression and significantly enhanced AMPK phosphorylation (Fig. 6a and b). In contrast, this expression pattern was again reversed by the co-transfection of shSirt1 (Fig. 6a and b). These results demonstrated that miR-122 suppressed Sirt1 expression, which further restrained the LKB1/AMPK signalling pathway.

## Discussion

NAFLD is characterized by excessive storage of lipid droplets in hepatic tissues, whose normal physiological functioning is thus gradually compromised (Dai et al., 2017b). As a prevalent disease worldwide, NAFLD has resulted in tremendous costs for the healthy system, and severe steatohepatitis can even progress to liver cirrhosis or hepatocellular carcinoma (HCC). In this study, we first found that miR-122 was upregulated in NAFLD hepatic tissues/cells, while Sirt1 was downregulated. After knocking down miR-122 in HepG2 or Huh-7 cells by the transfection of miR-122 inhibitor, excessive lipid deposition was effectively rescued, which could be attributed to the upregulation of Sirt1 and the activation of the LKB1/AMPK signalling pathway. These findings thus provide new insights into the pathogenesis, diagnosis and therapyof NAFLD.

A recent study demonstrated that miRNAs, such as miR-17, miR-20a, miR-20b and miR-122, were closely associated with the initiation and progression of NAFLD (Ye et al., 2018). Due to the intrinsic function of miRNAs in modulating the expression of target genes, current studies mainly focus on the roles of miRNAs in the regulation of downstream genes, especially those involved in lipid metabolism. As reported, miR-24 has been found to promote hepatic lipid accumulation by suppressing the expression of insulin-induced gene 1 (Ng et al., 2014). Early studies have shown that miR-122 is a liver-specific miRNA and is closely associated with liver-related diseases (Alizadeh et al., 2015). In the present study, we found that miR-122 expression was remarkably elevated in both NAFLD mice and FFA-treated hepatocytes as determined by qRT-PCR. This result was consistent with a previous report that showed that there was an evident correlation between high miR-122 expression and NAFLD incidence in obese children (Brandt et al., 2018). Furthermore, several studies also confirmed a positive association between circulating miR-122 and histopathological features in NAFLD patients (Jampoka et al., 2018; Ye et al., 2018; Akuta et al., 2016; Miyaaki et al., 2014). However, some previous studies showed that liver miR-122 levels were decreased while serum miR-122 levels were increased in human non-alcoholic steatohepatitis (Braza-Boils et al., 2016; Latorre et al., 2017; Sendi et al., 2018), which was not consistent with our results. We speculated that the different results might be due to the different experimental models and induction conditions of NAFLD, as well as the different severities of NAFLD patients. Overall, these previous studies and our results further substantiate the potency of miR-122 in the diagnosis and evaluation of NAFLD disease, although the underlying molecular mechanism still requires further investigation.

To identify the downstream target of miR-122 in our model, we first focused on Sirt1, which has been found to be downregulated in NAFLD patients (Castro et al., 2013). A previous study also suggested that restoring Sirt1 expressioncould become a promising therapeutic strategy for NAFLD (Colak et al., 2014) due to the probable association between Sirt1 and lipid metabolic genes, such as SREBP1, FASN, and SCD1 (Wang et al., 2017; Sun et al., 2015). We thus investigated the possible involvement of miR-122 in regulating Sirt1 and its effect on the expression of lipogenic genes. Both RIP and dual luciferase reporter assays confirmed that Sirt1 was a direct target of miR-122 whose expression was repressed through the binding of miR-122 to its 3′-UTR and consequential mRNA degradation. The knockdown of Sirt1, on the other hand, abolished the suppressive effect of miR-122 inhibitor on lipogenesis. Moreover, multiple other miRNAs, such as miR-9 (Ao et al., 2016) and miR-34a (Kim et al., 2015), also target Sirt1 and facilitate NAFLD progression via promoting lipogenesis. However, we first revealed that Sirt1 was a direct target of miR-122 and that miR-122 promoted NAFLD by directly inhibiting the expression of Sirt1.

NAFLD pathogenesis is closely related to lipid metabolic genes, including SREBP1, FASN, SCD1, ACC1 and ApoA5 (Lin et al., 2017; da Silva-Santi et al., 2016), whose expression are known to be regulated by distinct miRNAs. Until now, the role of miR-122 in modulating these genes and NAFLD progression has not been investigated. In this study, we further explored the functional effects of miR-122 in NAFLD. It was noted that miR-122 inhibition significantly decreased lipid accumulation and TG secretion in hepatocytes treated with FFA. In line with these results, one recent study reported that miR-122 played crucial roles in lipid droplet formation and TG secretion in hepatocytes under FFA-induced stress in culture medium (Wu et al., 2017). In addition, we examined the expression pattern of lipogenic genes in HepG2 and Huh-7 cells and found that they were prominently downregulated after transfection of the miR-122 inhibitor. These results showed that knockdown of miR-122 could alleviate NAFLD via suppressing the expression of lipid metabolic genes by directly upregulating Sirt1.

The LKB1/AMPK pathway is crucial for maintaining cellular homeostasis, especially for lipid metabolism (Lin et al., 2015). A previous study suggested that the LKB1/AMPK axis was involved in liver pathology since its activation could prevent the occurrence of liver diseases (Santamarina et al., 2015). Moreover, LKB1 is believed to be downstream of Sirt1 and plays an indispensable role in ameliorating NAFLD conditions (Jia et al., 2016). As reported, LKB1 could suppress the expression of various lipogenesis-related genes, such as FASN and adiponectin (Gormand et al., 2014). In our experiments, we found that FFA treatment could suppress the activity of the LKB1/AMPK pathway, while miR-122 knockdown potently re-activated this pathway. These results indicated that the Sirt1/LKB1/AMPK pathway was responsible for the function of miR-122 in regulating lipid metabolism in hepatocytes. In addition, emerging evidence has demonstrated that tumour growth factor-β (TGF-β) signalling, mammalian target of rapamycin complex 1 (mTOC1) signalling, Hedgehog signalling, c-Jun N-terminal kinase (JNK) signalling and MAPK signalling also participate in the regulation of lipid metabolism in the liver. However, only a few reports have explored the role of miR-122 in the modulation of these signalling pathways. It was reported that miR-122 and Sirt1 suppressed TGF-β signalling to affect liver cancer metastasis (Yin et al., 2016) and ameliorate renal fibrosis (Huang et al., 2014), respectively. However, the association between the miR-122-Sirt1 axis and these signalling pathways in lipid metabolism has not yet been reported and requires further investigation.

## Conclusions

In summary, this study found that miR-122 could repress the LKB1/AMPK signalling pathway via directly downregulating Sirt1, which then induced steatosis and lipogenesis in NAFLD. Therefore, knocking down miR-122 could re-activate the LKB1/AMPK axis, suppress lipid overproduction and alleviate NAFLD severity. These results revealed a novel function and detailed mechanism of miR-122 in regulating lipid metabolism in the liver, suggesting that miR-122 is a potential biomarker for NAFLD diagnosis and an appealing drug target for NAFLD therapy in the future.

## Abbreviations

ACC1: Acetyl-coA carboxylase
AMPK: AMP-activated protein kinase
ApoA5: Apolipoprotein A5
FASN: Fatty acid synthase
FFA: Free fatty acid
HCC: Hepatocellular carcinoma
HE: Hematoxylin-eosin
HFD: High fat diet
IHC: Immunohistochemistry
JNK1: c-Jun N-terminal kinase 1
LKB1: Liver kinase B1
NAD: Nicotinamide adenine dinucleotide
NAFLD: Non-alcoholic fatty liver disease
p-AMPK: AMPK phosphorylation
qRT-PCR: quantitative real-time PCR
RIP: RNA-immunoprecipitation
SCD: Standard chow diet
SCD1: Stearoyl-coA desaturase 1
shRNA: short hairpin RNA
Sirt1: Sirtuin 1
SREBP1: Sterol regulatory element-binding protein 1
TG: Triglyceride
TGF-β: tumor growth factor-β
mTOC1: mammalian target of rapamycin complex 1
